# The Role of the 21-Gene Recurrence Score^®^ Assay in Hormone Receptor-Positive, Node-Positive Breast Cancer: The Canadian Experience

**DOI:** 10.3390/curroncol29030163

**Published:** 2022-03-16

**Authors:** Mariya Yordanova, Saima Hassan

**Affiliations:** 1 Faculty of Medicine and Health Sciences, Université de Sherbrooke, Sherbrooke, QC J1K 2R1, Canada; mariya.yordanova@usherbrooke.ca; 2 Faculty of Medicine, Université de Montréal, Montréal, QC H3C 3T5, Canada; 3 Centre de Recherche du Centre Hospitalier de l’Université de Montréal (CRCHUM), l’Institut de Cancer de Montréal, Montréal, QC H2X 0A9, Canada; 4 Division of Surgical Oncology, Department of Surgery, Centre Hospitalier de l’Université de Montréal (CHUM), Montréal, QC H2X 0C1, Canada

**Keywords:** Oncotype DX Recurrence Score assay, hormone receptor-positive breast cancer, node-positive, decision impact, cost-utility, Canada

## Abstract

The management of patients with hormone receptor-positive breast cancer has changed dramatically with use of the 21-gene Recurrence Score^®^ (RS) Assay. While the utility of the assay was initially demonstrated among node-negative patients, recent studies have also demonstrated the assay’s prognostic and predictive value in node-positive patients. In Canada, the RS assay is reimbursed by provincial health insurance plans, but not all provinces have approved the use of the assay for patients with node-positive disease. Here, we provide an overview of the clinical factors that influence physician recommendation of the RS assay and, alternatively, the impact of the RS assay on patient treatment decisions in Canada. We performed a comprehensive review of the impact of the assay upon physician treatment decisions and cost in node-positive breast cancer patients within Canada and other countries. Furthermore, we evaluated biomarkers that can predict the RS result, in addition to other genomic assays that predict recurrence risk among node-positive patients. Overall, the 21-gene RS assay was shown to be a cost-effective tool that significantly reduced the use of chemotherapy in node-positive breast cancer patients in Canada.

## 1. Introduction

The prognosis of patients with breast cancer has improved in recent years mainly due to improvements in screening and new therapeutic options [1,2]. However, our understanding of prognostic and predictive biomarkers has also changed our approach in administering chemotherapy in the adjuvant setting, thus, allowing us to better select treatments for patients. Decisions for administering chemotherapy have historically been based on clinical and pathological features that prognosticate breast cancers. These features include younger age, tumor size, tumor grade, hormone and growth factor receptor status - including estrogen receptor (ER), progesterone receptor (PR), human epidermal growth factor receptor 2 (HER2), and regional lymph node involvement [3,4,5,6]. Tumor involvement of axillary lymph nodes (node-positivity) has been considered the strongest prognostic marker for breast cancer [7]. However, studies have demonstrated limitations in the prognostic role of lymph nodes due to a lack of sensitivity and specificity. About one-third of node-negative breast cancer patients develop distant metastasis, whereas one-third of node-positive patients remain free of distant metastasis ten years after local therapy [8,9,10]. 

Over the past two decades, a multi-gene expression test of the primary tumor has revolutionized the manner in which clinicians administer adjuvant chemotherapy in ER-positive breast cancer patients. The Oncotype DX Breast Recurrence Score^®^ (RS) assay (Exact Sciences) is a multi-gene reverse transcription-polymerase chain reaction (RT-PCR) test that analyzes the expression of 21 genes (16 breast cancer and five reference genes). An algorithm is then used to calculate a RS on a scale of 0–100 to determine risk of distant relapse. Originally derived from a cohort of node-negative patients in 2004, Paik et al. divided the continuous RS into three categories: low-risk (RS < 18), intermediate-risk (RS, 18–30), and high-risk (RS > 30), with corresponding rates of distant recurrence at 10 years at 6.8%, 14.3%, 30.5%, respectively, amongst patients receiving tamoxifen only [11]. However, the Trial Assigning Individualized Options for Treatment (TAILORx) redefined the cutpoints to minimize the potential for undertreatment [12]. Here, the risk categories were defined as: low (RS < 11), intermediate (RS 11–25), and high (RS > 25).

The TAILORx trial was designed to prospectively validate the RS assay in 10,273 women with hormone receptor (HR)-positive, HER2-negative, axillary node-negative breast cancer, wherein patients with a RS 11–25 were randomized to receive chemoendocrine versus endocrine therapy [13]. Amongst all patients, endocrine therapy was comparable to the chemoendocrine therapy group, in terms of freedom from disease recurrence at a distant or local regional site as well as overall survival. However, in patients ≤ 50 years with RS 16–25, there was a 3.4–8.7% difference in freedom from disease recurrence at distant or local regional site at nine years for patients receiving chemoendocrine therapy versus endocrine therapy but comparable responses in patients with RS < 16. Overall, this study demonstrated that the assay can identify up to 85% of women from whom adjuvant chemotherapy can be omitted, particularly for women > 50 years with a RS < 26 and those ≤50 years with a RS < 16.

The prognostic role of the RS result has also been assessed in cohorts consisting of both node-negative and node-positive patients. Amongst postmenopausal patients who were treated with either tamoxifen or anastrozole alone in the Arimidex, Tamoxifen, Alone or in Combination (ATAC) trial [14], the 9-year risk of distant recurrence for node-negative patients was 4%, 12%, and 25%, for the RS low, intermediate, and high-risk patients, respectively; and for node-positive patients, was 17%, 28%, and 49%, respectively. In pre- and postmenopausal women with zero to three positive nodes receiving chemotherapy in the Eastern Cooperative Oncology Group (ECOG) E2197 trial [15], the RS result was also a strong prognostic biomarker for locoregional or distant recurrence in patients with both node-negative (*p* < 0.001) and node-positive disease (*p* < 0.001). This suggests that the RS is prognostic in node-negative and node-positive patient cohorts with otherwise similar clinicopathological and treatment parameters.

Although several retrospective studies supported the use of the 21-gene assay in HR-positive, HER2-negative, node-positive breast cancer patients [16], it was only recently that a prospective trial reported the results of the assay in a population of one to three positive lymph node patients [17]. In the RxPONDER trial, 5083 women with a RS ≤ 25 were randomized to receive either chemoendocrine therapy or endocrine therapy alone. Amongst postmenopausal women, invasive disease-free survival at 5 years was comparable at ~91–92% for patients receiving chemoendocrine versus endocrine therapy, demonstrating no added benefit from adjuvant chemotherapy. In the premenopausal group, invasive disease-free survival at 5 years for the endocrine-only and the chemoendocrine treatment group were 89.0% and 93.9%, respectively (hazard ratio (HR) 0.58; 95% CI, 0.39 to 0.87, *p* = 0.009), showing significant benefit from chemotherapy in this subset of the population. However, it is important to note that, when using the prespecified RS cutpoint range of 0–13, there was no statistically significant difference between the endocrine and chemoendocrine group for the premenopausal women (HR 0.51; 95% CI, 0.25 to 1.04, *p* = 0.062). Therefore, the RxPONDER trial established the expanded utility of the 21-gene assay to postmenopausal node-positive patients.

Accordingly, recent evidence supports the need to integrate the RS assay in the management of node-negative and node-positive patients to guide the use of chemotherapy in the adjuvant setting. In Canada, the provincial government is responsible for the reimbursement of the assay. While approvals have been made in all ten provinces for node-negative patients, provinces are still in the process of acquiring approval for patients with node-positive disease. This has resulted in several provincial studies evaluating clinical utility and cost-effectiveness of the RS assay. Here, we first provide a brief overview of the patient and physician perspective of the RS assay. We then review the clinical utility of the RS assay in guiding physician’s decisions and its cost-effectiveness across different provinces in Canada and other countries. Moreover, we evaluate the role of predictive biomarkers of the RS result, and finally, the evidence supporting the use of other genomic assays in node-positive breast cancer patients.

## 2. Use of the RS Assay in Canada

Two Canadian survey studies evaluated the clinicopathological features that influenced physician use of the RS assay and the patient perception of the assay [18,19]. In a survey of 47 medical oncologists [18], the clinicopathological factors considered to strongly influence the use of the RS assay in at least 60% of respondents included ER/PR status, lymph node status, patient preference, and patient request. Features that were less commonly considered to strongly influence use of the RS assay included tumor size, lymphovascular invasion, Adjuvant! Online risk score, and menopausal status. The assay was more frequently used in patients aged 40–65, grade 2 tumors (versus grade 1 or 3), patients with few comorbidities, HR-positive status, and node-negative or microscopic metastasis (versus ≥1 positive node), which is consistent with public funding criteria and available data at the time of the survey, prior to the RxPONDER trial. The most cited reason for using the assay among responders was to avoid unnecessary chemotherapy, with usage primarily among patients with intermediate risk factors.

Interestingly, Marshall et al. conducted a discrete choice experiment survey among early-breast cancer patients to assess their perspective on the benefits and risks of chemotherapy treatment based on the use of gene expression profiling (GEP), such as the RS assay [19]. Among the 1004 Canadian women who responded, about 80% knew someone who had chemotherapy for cancer; however, only 5% knew someone having received a GEP test. The most important factor for patient decision was the GEP test score that indicated benefit of the chemotherapy. Other factors of importance were the risk of relapse, trust in oncologist, and the side effects of the chemotherapy, with permanent side effects being the next most influential aspect and temporary side effects being the least. Low, medium, and high-risk scenarios were created based on the clinical risk of recurrence and GEP test score. Thirty-three percent of patients selected chemotherapy in a low-risk scenario, 55% in a moderate-risk scenario and 78% in a high-risk scenario. This demonstrates that recurrence risk based on physician recommendation and GEP test result play an important role in influencing chemotherapy treatment decisions of patients.

## 3. Decision–Impact of the RS Assay

We will first review the decision-impact of the RS assay in HR-positive, HER2-negative, and node-positive breast cancer patients upon physician treatment decisions in individual provinces in Canada, followed by a review of global experiences (Table 1). 

The clinical utility of the RS assay on physician treatment decisions was evaluated in a prospective, multicenter study in a cohort of 84 patients from British Columbia [20]. Patients were recruited from 2015–2017. Questionnaires regarding decision treatments were administered to physicians after patient-physician consultations prior to and post-results of the RS assay. Treatment recommendation changed from chemoendocrine therapy to endocrine therapy alone for 49% of patients and 4% of recommendations changed from endocrine therapy to chemoendocrine therapy, and so the net reduction in the use of chemotherapy was 45%. Amongst patients with RS < 18, the net reduction in the use of chemotherapy was 55%, while patients with a RS result between 18–30 demonstrated a net reduction in the use of chemotherapy of 33%. This is suggestive of the clinical utility of the test in both low-risk and intermediate-risk RS groups.

Two studies evaluated the impact of the RS assay amongst node-positive patients in Ontario. Richardson et al. conducted a prospective online survey for collection of classical pathological and clinical characteristics pre- and post-RS test from 2016–2017 [21]. Data was collected from 12 centers for a total of 176 cases. In this cohort, 71% of patients were postmenopausal, and 69% were single-node positive. The RS distribution was as follows: 64% were low-risk (RS < 18), 28% were intermediate-risk (RS 18–30), and 9% were high- risk (RS > 30). Treatment recommendations pre- and post-RS result demonstrated a 51% net reduction in the usage of chemotherapy as adjuvant treatment (a total of 148 patients (84%) had treatment recommendation for chemoendocrine therapy pre-test, which decreased to 59 patients (34%) post-test).

Torres et al. evaluated the impact of the 21-gene RS assay on physician’s clinical decisions/recommendations on adjuvant chemoendocrine therapy as well as the level of confidence in said treatment decision also among node-positive patients in Ontario [22]. Questionnaires were used to capture physician’s recommended treatment plan, in addition to physician and patient levels of confidence both prior to and post-results of the RS assay. Between 2014–2016, 72 patients were recruited, of which 55% of tumors were low-risk (RS < 18), 36% were intermediate-risk (18–30), and 9% were high-risk (RS > 30). Overall, chemotherapy recommendation decreased by 27% (79% pre-assay versus 52% post-assay). Since 42% of patients ultimately received chemotherapy, this represented an actual reduction of chemotherapy use of 37%. The most significant change was in the low-risk RS group, wherein there was a 47% change in recommendation from chemoendocrine to endocrine therapy. The level of confidence for physicians and patients increased in 49% and 54% of cases, respectively.

Our group conducted a prospective, multicenter study in Quebec to partly evaluate the impact of the 21-gene RS assay in treatment decisions [23]. Physicians completed a questionnaire regarding their treatment decision pre- and post-availability of RS result. 70 patients were enrolled in 2018–2019. Eighty-one percent of the patients were ≥50 years, and 64.3% of the patients had one positive lymph node. Using cutpoints from the TAILORx trial, the RS distribution was as follows: 18% were low risk (RS < 11), 48% were intermediate-risk (RS 11–25) and 4% were classified as high-risk (RS > 25). There was a 72.2% reduction in physician recommendation in chemotherapy for patients with RS < 11 and 70.5% for patients with a RS between 11–25. The reduction in chemotherapy occurred in 73.3% of patients with one positive node, and 56.0% for patients with two or three positive nodes. Overall, there was a 67.1% decrease in chemotherapy recommendation for all patients (90.0% versus 22.9% pre- and post-assay). Therefore, in Canada, use of the RS assay resulted in a reduction in chemotherapy use ranging from 27–67%.

Studies from around the globe have demonstrated a similar significant impact of the RS assay on adjuvant therapy recommendations in node-positive tumors. Here, we selected decision–impact studies which included cohorts of more than 50 node-positive patients. In the following three studies, patient recruitment occurred from 2006–2015. In an Australian study of 122 node-positive patients, the recommendation for chemo-hormonal therapy decreased from 65% to 23% post-assay, leading to a reduction of 42% (*p* < 0.01) [24]. In a study conducted in Germany, 122 node-positive patients were also recruited [25]. Treatment recommendation changed from chemoendocrine therapy to endocrine therapy alone for 37% of patients. Physician confidence increased in 46% of node-positive cases. In a UK study, amongst a cohort of 65 node-positive patients, a 69.2% reduction in the use of chemotherapy was reported (with 30.8% of patients recommended chemotherapy post-assay) [26]. 

Studies that recruited patients in more recent years, from 2015–2019, also demonstrated a similar reduction in chemotherapy recommendation [27,28,29]. In a prospective multicenter study from Latin America comprising 131 node-positive patients, a 34% absolute reduction in the use of chemotherapy (63% pre-assay versus 28% post-assay) was observed [27]. Three recent prospective Italian studies demonstrated reductions in chemotherapy recommendations of 32% (50% pre-assay versus 27% post-assay) in 523 micrometastatic and node-positive patients [29], 28% (55% pre-assay to 27% post-assay) in 99 node-positive patients [28], or 18% in 127 node-positive patients [30]. In a Brazilian study, with a cohort of 58 node-positive patients that were recruited after the release of the TAILORx results, there was a 66% reduction in chemotherapy recommendation (100% pre-assay versus 34% post-assay) [31]. Therefore, the extent of reduction in chemotherapy recommendation from global studies ranged from 18–69%, and was fairly consistent with patients recruited throughout 2006–2019.

**Table 1:** Summary of decision impact studies in node-positive patients.

Author, Country, Year of Study	Dates of Enrollment	Single or Multicenter Study, Retro/Prospective	No. of Node- Positive Patients	21-Gene Recurrence Score Stratification Groups	Reduction of Chemotherapy Recommendation in Node-Positive Patients
L. Chin-Lenn et al. [24], Australia, 2018	2006–2014	Multicenter, retrospective	122	0–17, 18–31 >31	42% reduction
W. Eiermann et al. [25], Germany, 2013	June 2010– April 2011	Multicenter, prospective	122	NA	37% reduction
J. Loncaster et al. [26], UK, 2017	May 2012– March 2015	Single-institution, prospective	65	0–17, 18–30, 31–100	69% reduction
S. Torres et al. [22], Ontario, Canada, 2018	October 2014– May 2016	Single-institution, prospective	71	0–17, 18–30, 31–100	27% reduction in recommendation, 37% reduction in use of chemotherapy
N. LeVasseur et al. [20] British Columbia, Canada, 2021	December 2015– January 2017	Multicenter, prospective	84	0–17, 18–30, 31–100	45% net reduction
H. Gomez et al. [27], Latin America, 2021	March 2015– December 2019	Multicenter, prospective	131	0–17, 18–30, 31–100, and 0–10, 11–25, 26–100	39% reduction
F. Cognetti et al. [29], PONDx, Italy, 2021	February 2016– December 2017	Multicenter, prospective	523	0–17, 18–30, RS 31–100, and 0–25, 26–100	32% reduction
M. Dieci, et al. [28], ROXANE, Italy, 2019	January 2017– February 2018	Multicenter, prospective	99	0–10, 11–25, 26–100	28% reduction
A. Zambelli et al. [30], BONDX, Italy, 2020	January 2017– August 2018	Multicenter, prospective	127	0–17, 18–30, 31–100, and 0–10, 11–25, 26–100	18% reduction
S. Hassan et al. [23], Quebec, Canada, 2020	March 2018– September 2019	Multicenter, prospective	70	0–17, 18–30, 31–100, and 0–10, 11–25, 26–100	67% reduction
A. Mattar, et al. [31], Brazil, 2021	August 2018– April 2019	Multicenter, prospective	58	0–10, 11–25, 26–100	66% reduction

## 4. Cost Utility of the RS Assay

In this section, we will review the impact of the 21-gene RS assay on treatment costs, first within Canadian provinces and then amongst other countries in North America and Europe. 

Hannouf et al. compared the cost effectiveness of the 21-gene RS assay to the current Canadian clinical practice in postmenopausal women with HR-positive and lymph-node positive early-stage breast cancer [32]. To compare the two different treatment guiding strategies, the authors developed a decision analytic model to estimate the lifetime health and economic consequences. The RS assay-based strategy determines the risk classification of a patient (low, intermediate, or high), and treatment decision is established (chemoendocrine therapy or endocrine therapy alone). The current clinical practice classifies all women as high-risk requiring adjuvant chemotherapy. The model demonstrated an increase of 0.08 quality-adjusted life-year (QALY) when using the RS assay compared to current clinical practice, as well as an increase in cost of $36 (Canadian dollars) per person resulting in an incremental cost-effectiveness ratio (ICER) of $464/QALY gained.

Lamond et al. similarly looked at the cost-utility of the RS assay this time in node-positive versus node-negative patients, HR-positive breast cancer [33]. A state-transition model was created to calculate cumulative costs and QALY over a 25-year period. The chemotherapy utilization proportion was derived from a Nova Scotia Canadian population-based cohort, local unit cost and recent literature. An RS-guided approach was associated with incremental costs of $2585 and $864, QALY gains of 0.27 and 0.06, and cost-utility of $9591 and $14,844 per QALY gained for node-negative and node-positive disease, respectively. This data suggests that an RS-guided decision for chemotherapy is a cost-effective strategy for both node-negative and node-positive breast cancer.

Masucci et al. likewise evaluated the functional utility of the RS assay in node-positive, HR-positive and HER2-negative breast cancer patients in Ontario by developing a Markov model to determine the cost and QALY for a patient’s lifetime [34]. Patients’ evolution and outcomes were derived from published clinical trials, and costs from published Canadian sources. The RS assay was shown to be less costly ($432 less) and more effective (0.22 QALYS) than the current standard of care over a lifetime, providing 0.17 life-years gained. Thus, this study supports the concept that the 21-gene RS assay is a cost-effective approach in patients with node-positive early breast cancer.

As part of our prospective multicenter study in Quebec, we evaluated the impact of the 21-gene RS assay on chemotherapy use in node-positive breast cancer patients, as well as its effect on costs. We demonstrated a decrease in the total cost of chemotherapy by 69.9% per patient following assay results (pre-RS mean, $3968 CAN; versus post-RS mean, $1196 CAN), supporting the overall cost savings associated with the RS assay [23].

Several studies from around the world have also demonstrated the economic benefits of the RS assay. In an Irish study with 963 HR-positive node-negative breast cancer patients, a 62.5% reduction in chemotherapy use was identified post-assay result. This led to a savings of €4,254,110 (Euros) in treatment cost. Despite concerns regarding the cost of the 21-gene test, even when factoring the cost for all patients, a net overall savings of €1,191,770 was still attained [35]. A study from the United States investigated the cost-effectiveness of the RS assay by creating state-transition models that estimate cost and QALY gained over lifetime in a cohort of 2245 HR-positive, HER2-negative and node-negative breast cancer patients. The authors reported a differential ICER for the RS assay across clinical risk groups ranging from $124,600 per QALY in the low-risk group, to $28,700 per QALY in the intermediate-risk group, and $15,700 per QALY in the high-risk group. When grouping all patients together, the ICER of RS assay was $62,200 per QALY. These results demonstrate that the RS tool is cost-effective, particularly in intermediate and high-risk groups [36].

Cost-effectiveness has similarly been demonstrated in mixed cohorts of patients with node-negative and node-positive disease. Eiermann et al. demonstrated an increase in the life-expectancy by 0.06 years and a reduction in cost with the RS assay by €561 when compared to the standard of care [25]. Loncaster et al. reported a cost savings of GBP 266,427 (Great Britain Pounds) when using the RS assay to reduce chemotherapy utilization in patients [26]. The Italian BONDX study consisted of 394 node-negative and node-positive patients, which demonstrated a total budget reduction of €81,017 from sparing chemotherapy in patients as the proportion of patient with chemotherapy recommendation was reduced from 24.6% to 15.2% post-21-gene RS testing [30]. A cost-utility study performed in Spain in a cohort of 401 patients also supported the cost-effectiveness of the intervention among a cohort with node-positive breast cancer patients as each QALY gained cost less than €25,000 [37]. 

Lastly, Berdunov et al. developed a Markov-based model calculating the cost-effectiveness of RS assay before and after the RxPONDER trial results in a cohort of postmenopausal women with HR-positive, HER2-negative and node-positive disease [38]. The probability of the RS assay of having a cost-effectiveness of €20,000/QALY was 97.9% with inclusion of the RxPONDER trial results, compared to 51.5% in the previous model. Furthermore, updating the model showed a reduced cost of chemotherapy and higher QALYs compared to clinical risk tools. Taken together, the RS assay was associated with a significant reduction in cost, with cost-utility demonstrated in node-negative and node-positive populations, including in Canada and several other countries.

## 5. Predicting Oncotype DX RS Results

Due to concerns of cost and delay in obtaining results, a few Canadian studies evaluated clinicopathological factors that may be predictive of the Oncotype DX RS result.

In a retrospective cohort study with 425 node-negative breast cancer patients from Quebec, clinicopathological data was correlated with available RS results [39]. Patients with PR-negative and histologic grade 2 tumors were more likely to have an intermediate or high RS versus low RS result, based on Paik et al.’s cutpoints [11]. The authors further explored the impact of the RS result on the utilization of adjuvant chemotherapy. While 92.5% of patients with a high RS result received chemotherapy, only 42.6% of patients received chemotherapy in the intermediate RS group, emphasizing the impact of accurate risk classification. Nevertheless, while the study suggested that histologic grade and PR status may be potential predictors of the RS, additional studies are still warranted.

In a cohort of 201 breast cancer patients with node-negative or micrometastatic disease, an Ontario group of researchers used four tools to either compare their prognostic impact or predictive potential of the actual RS result [40]. These tools included: (1) the PREDICT tool, an online calculator (available at https://breast.predict.nhs.uk/) [41]; (2) the simplified risk score, which uses ER, PR, tumor size, nuclear grade and histologic grade to calculate a score ranging from 0–21; (3) the Tennessee prognosticator, which is based on age, tumor size and grade, PR status, presence of lymphovascular invasion, and histologic subtype; and (4) GR-PR, derived from the study group themselves, which attributes a score of 0–2 based on the presence of a grade 3 tumor or PR staining of any intensity in ≤20% of tumor cells. The authors used the PREDICT tool to compare the 10-year overall survival with the 10-year distant relapse free-survival obtained by the RS result; however, no correlation was identified. The three additional tests demonstrated comparable sensitivity, specificity, and positive and negative predictive value. The simplified risk score accurately classified 100% patients in their respective low or intermediate-risk RS with a value < 7, while a high score of >12 only correctly classified 19% of patients. As for the Tennessee predictor, the percentage of patients correctly classified within a particular interval was low, offering limited confidence in the tool, except for the <18 cutpoint. The GR-PR score was a strong predictor of tumors either having a RS < 18 or >30, however, was less successful with the TAILORx-defined categories. Overall, while these tools demonstrated promising results, they also require further validation.

Additionally, an Alberta-based group evaluated Ki67—a cancer cell proliferation marker—as a predictive biomarker of the RS result in a retrospective study of 328 node-negative breast cancer patients [42]. The utilization of Ki67 has, in the past, raised issues of variability due to selection bias introduced when scoring tumor regions. Therefore, the authors developed an automated Ki67 scoring method with a whole-slide analysis, which was highly concordant with manual scoring by pathologist (Pearson’s r = 0.909) and by users (Pearson’s r = 0.984). High Ki67 indices were found to correlate with RS groups (low versus high, *p* < 0.001). Moreover, when Ki67 was incorporated in a random forest machine learning model, high and low-risk patients from the RS assay were identified with 97% accuracy, 98% sensitivity, and 80% specificity. Consequently, Ki67 scores may play an important role in predicting recurrence risk in breast cancer patients.

Similarly, a multivariable model, called the Magee Decision Algorithm (freely available online [43]), was derived to better predict the RS result mainly in the context of node-negative early breast cancer patients. The Magee Decision Algorithm is derived from three equations (Magee 1/2/3) that include ER/PR, H-score (sum of the product of percentage of cells and staining intensity), grade, tumor size, and Ki67 [44]. Interestingly, the Magee 3 equation was shown to be a robust screening tool, in which 52% of 212 patients could forego the Oncotype DX RS test in a study from Robertson and colleagues from Ottawa, Canada [45]. In a larger cohort of 2196 patients, the Magee Decision Algorithm in combination with a mitosis score predicted which patients could forego RS testing with an accuracy of 95% [46]. Although the predictive value of this test needs to be specifically evaluated in the context of node-positive patients, this approach demonstrates potential to improve the overall cost-effectiveness of the RS assay [46,47].

## 6. Other Genomic Tests

The Oncotype DX RS Assay is supported with Level 1 evidence for use in node-negative and postmenopausal node-positive patients by the National Comprehensive Cancer Network (NCCN) [48]. While the 21-gene RS assay is the most commonly used genomic test for early breast cancer patients in Canada [49], we will briefly discuss some of the other available genomic tests and their role in node-positive disease.

### 6.1. Mammaprint

Mammaprint (Agendia) is a 70-gene assay that was originally described by van’t Veer et al. in a selected group of younger patients with node-negative disease in 2002 [50,51,52]. Although the GEP was originally conducted from frozen tissue, the assay has since been modified for use with formalin-fixed paraffin-embedded (FFPE) tissue. Furthermore, Mammaprint has been validated in several studies, including large cohorts with node-positive disease [52]. In particular, 9-year follow-up from the MINDACT study was recently reported amongst patients with low genomic risk inferred by Mammaprint yet high clinical risk [53]. Amongst 658 node-positive patients, the 8-year distant metastasis-free survival differed by only 1.3% in patients who did and did not receive chemotherapy, further confirming the prognostic utility of this test in this cohort. Mammaprint has been endorsed by the American Society of Clinical Oncology (ASCO), European Society of Medical Oncology (ESMO), and Ontario Health in the context of HR-positive, HER2-negative tumors and node-positive disease [52,54]. Since formal reimbursement was only available in Ontario as of October 2021 in the context of node-negative disease [55], we have yet to determine its true clinical utility in Canada.

### 6.2. Prosigna, Endopredict, and Breast Cancer Index

Prosigna (Veracyte) is a commercially available assay derived from the Prediction Analysis of Microarray 50 (PAM50) 50-gene classifier into the four intrinsic subtypes, including luminal A, luminal B, HER2-enriched, and basal-like [56]. The Prosigna Risk of Recurrence (RoR) score uses Nanostring nCounter^TM^ technology to digitally quantify gene expression from FFPE tissue, creating a continuous score between 0–100, which provides a 10-year risk of distant recurrence. Endopredict (Myriad Genetics) is a 12-gene test that uses RT-PCR to calculate a risk score and in combination with clinical parameters has been termed the EPclin score. The Breast Cancer Index (BCI) (Biotheranostics) also uses RT-PCR for seven genes, including a two-gene ratio of the anti-apoptotic homeobox B13-to-interleukin 17B receptor, and five proliferation genes (the Molecular Grade Index). This assay was designed to determine the added benefit of extending endocrine therapy from 5 to 10 years [52,57].

Many retrospective studies have identified prognostic utility of these assays in post-menopausal early breast cancer patients with node-negative and node-positive disease [52,57,58,59,60]. While these tests have been endorsed by Ontario Health for node-negative patients [54], their role in the node-positive context has yet to be clearly established. While ASCO does not recommend their use in node-positive disease due to either inconsistent data regarding choice of cutpoint or insufficient quality of evidence [61], NCCN’s recommendation is based on level 2A evidence [48].

## 7. Discussion

The Oncotype DX RS assay first demonstrated its role amongst node-negative HR-positive early breast cancer patients and is reimbursed in ten Canadian provinces in this setting [49]. Intuitively, the RS assay was a logical approach to further risk-stratify node-positive patients to better select which patients would benefit from chemotherapy. However, the manner in which the RS assay has infiltrated the node-positive arena is rather fascinating. As evidenced from large retrospective registry studies [62,63,64], clinicians were ready to embrace the RS assay in their decision-making process amongst node-positive patients prior to the publication of results from large prospective validation trials. Thus, clinicians were perhaps already questioning two concepts: first, that there can be a prognostic biomarker stronger than lymph node positivity, and second, that not all node-positive patients need to be treated with chemotherapy. Indeed, one of the greatest sources of variability in the decision-impact studies is that physician recommendations for chemotherapy prior to RS testing were between 50–100%, suggesting that the clinical rationale for chemotherapy recommendation was not consistent in node-positive patients. It is plausible that improved access to the RS assay among node-positive breast cancer patients can decrease such variability in physician treatment recommendations.

We reviewed the clinical utility of the RS assay by focusing on Canadian studies and comparing with studies from around the world. Regardless of the patient cohort size and country of origin, a reduction in chemotherapy recommendation and use was demonstrated across all trials with moderate variations in magnitude of reduction. Although the magnitude of reduction of chemotherapy recommendation did not increase progressively with advancing years of patient accrual, it is likely that with the release of TAILORx and RxPONDER results, we can envision an even greater reduction in the use of chemotherapy [29].

We also evaluated cost utility, and several studies demonstrated the immediate and future cost benefit of the RS assay. While many of the studies evaluated node-negative or mixed populations, it is probable that greater reductions in cost will be observed in the future with the node-positive population since the prospective validation from the RxPONDER trial. Furthermore, greater cost benefits may be observed in cohorts that would have otherwise had a high proportion of chemotherapy use prior to the RS assay [33]. Therefore, the true cost benefit of the RS assay is yet to be elucidated.

Several surrogate biomarkers have emerged to predict the RS result. Despite suggestive results, no single biomarker or online tool demonstrated an equivalent prognostic or predictive potential as the RS assay in a prospective manner. In addition to the 21-gene RS assay, several other genomic tools can assist with risk stratification and treatment decisions in early breast cancer. Comparisons of these genomic assays in patients, including node-positive disease, suggested that each test had independent prognostic value or risk estimates, implying that the assays could not replace one another [65,66,67]. However, in the context of breast cancer patients with one to three positive nodes, the strongest evidence only supports the use of either the 21-gene RS or Mammaprint assays for adjuvant treatment decisions.

## 8. Conclusions

Overall, the TAILORx and RxPONDER trials established the significance of the RS assay in foregoing chemotherapy in early breast cancer, amongst both node-negative patients and postmenopausal node-positive patients. Survey studies amongst Canadian physicians identified clinical and tumor features which strongly influenced physician recommendation of the RS assay and the significance of a genomic test in influencing treatment decisions in node-negative patients. In node-positive patients, decision–impact studies showed that the RS assay reduced chemotherapy recommendations by 27–67% in Canada and 18–69% in studies from the world-over. Important overall savings in cost were identified both immediately and in terms of gains in long-term QALY. To further evaluate other cost-reducing approaches, several studies were conducted to examine surrogate biomarkers that can predict RS results. Although interesting results were shown with Ki67-based analysis and online tools, additional prospective studies are required to establish these biomarkers in the node-positive cohorts. Furthermore, various genomic assays have been tested in node-negative and node-positive patients. However, the 21-gene RS assay has strong prognostic and predictive value from prospective studies and is the most utilized assay amongst early breast cancer patients with node-positive disease in Canada. Taken together, the RS assay has demonstrated an important role in changing the patient-physician treatment decisions to provide personalized therapy amongst hormone receptor-positive node-positive breast cancer patients.

## Data Availability

Not applicable.
